# Pheno‐Deep Counter: a unified and versatile deep learning architecture for leaf counting

**DOI:** 10.1111/tpj.14064

**Published:** 2018-09-11

**Authors:** Mario Valerio Giuffrida, Peter Doerner, Sotirios A. Tsaftaris

**Affiliations:** ^1^ Institute for Digital Communications School of Engineering University of Edinburgh Thomas Bayes Road EH9 3FG Edinburgh UK; ^2^ IMT School for Advanced Studies Piazza S. Francesco 19 55100 Lucca Italy; ^3^ School of Biological Sciences University of Edinburgh Mayfield Road Edinburgh EH9 3JR UK; ^4^ The Alan Turing Institute 96 Euston Road London NW1 2DB UK

**Keywords:** image‐based plant phenotyping, machine learning, deep learning, leaf counting, multimodal, night images

## Abstract

Direct observation of morphological plant traits is tedious and a bottleneck for high‐throughput phenotyping. Hence, interest in image‐based analysis is increasing, with the requirement for software that can reliably extract plant traits, such as leaf count, preferably across a variety of species and growth conditions. However, current leaf counting methods do not work across species or conditions and therefore may lack broad utility. In this paper, we present Pheno‐Deep Counter, a single deep network that can predict leaf count in two‐dimensional (2D) plant images of different species with a rosette‐shaped appearance. We demonstrate that our architecture can count leaves from multi‐modal 2D images, such as visible light, fluorescence and near‐infrared. Our network design is flexible, allowing for inputs to be added or removed to accommodate new modalities. Furthermore, our architecture can be used as is without requiring dataset‐specific customization of the internal structure of the network, opening its use to new scenarios. Pheno‐Deep Counter is able to produce accurate predictions in many plant species and, once trained, can count leaves in a few seconds. Through our universal and open source approach to deep counting we aim to broaden utilization of machine learning‐based approaches to leaf counting. Our implementation can be downloaded at https://bitbucket.org/tuttoweb/pheno-deep-counter.

## Significance statement

Machine learning could become a valuable tool in extracting phenotypic traits, such as leaf count, from images of plants. We propose a deep neural network to count leaves of rosette‐type plants. Our approach achieves outstanding results in different settings (including different species and cultivars) and can combine multiple imaging sources. By making it openly available to the community we hope to further stimulate large‐scale analysis in plant phenotyping and help towards relieving the analysis bottleneck.

## Introduction

Image‐based plant phenotyping has recently become a valuable tool for quantitative analysis of plant images. However, its rapid expansion has highlighted the need for reliable software solutions with the power to analyze data efficiently (Gehan *et al*., 2017). While the bottleneck was previously thought to be the acquisition of imaging data (i.e. the hardware; Furbank and Tester, 2011), it has recently shifted to a lack of reliable software (and algorithms) (Minervini *et al*., 2015a), due to the sheer number of imaging data that need to be analyzed to extract quantitative plant traits. Machine learning has been proposed as a suitable solution to effectively extract plant traits (Singh *et al*., 2016; Tsaftaris *et al*., 2016).

Leaf count is an important plant trait and is directly related to the development stage of the plant (Boyes *et al*., 2001) and its flowering time (Chien and Sussex, 1996), yield potential (Kouressy *et al*., 2008) and health (Rahnemoonfar and Sheppard, 2017). Until recently, leaf counting was treated as a by‐product of leaf segmentation with deterministic image processing techniques. For example, most of the methods in the seminal collation study of leaf segmentation (Scharr *et al*., 2016) perform the following processing steps: first, they isolate the plant from the outer background (per plant segmentation) and then apply certain heuristics to delineate each leaf (per leaf segmentation). For example, IPK (Pape and Klukas, 2015a) uses color images to extract geometrical representations of the isolated plant to find suitable split points to separate each leaf, relying on assumptions about plant shape and structure (e.g. reduced leaf overlap and visible long leaf blades). Aksoy *et al*. (2015) employed a clustering algorithm to delineate leaves on near‐infrared (NIR) images of tobacco plants, where the per leaf segmentation was further improved using shape models. In general, the main drawback of these deterministic approaches is that such heuristics may fail when they encounter new data, reducing their applicability to different setups: for example, the performance of IPK drops by over 20% when this algorithm is applied to tobacco plants, where blade overlap is significant (Pape and Klukas, 2015a). Hence, users are faced with a dilemma: either to adapt the many parameters of these methods or to derive completely new ad hoc heuristic methods suitable for new imaging settings and plant species.

Machine learning is an alternative approach: rather than having users adapt methods, they instead provide their expertise by doing tasks they know well and have always been doing – phenotyping by observation. For example, in the context of leaf counting, a user may give observations (referred to as annotations in machine learning) either by delineating each leaf (a time‐consuming finely grained annotation) or by giving the location of each leaf (less time‐consuming) or just the total number of leaves in each plant. It is then the task of the machine‐learning algorithm to learn from such examples (known as the training set), i.e. the combination of images and corresponding annotations.

Romera‐Paredes and Torr (2016) and Ren and Zemel (2017) proposed very sophisticated deep neural network models that, given a training set of images and precise leaf delineations, learn per leaf segmentation and leaf count. They both evaluated their general method in plant images of wild‐type Arabidopsis based on an open dataset (Minervini *et al*., 2016). However, the collection of such finely grained annotations is tedious and time‐consuming (Minervini *et al*., 2015b; Minervini *et al*., 2017), particularly when one must annotate data of significant diversity to account for large leaf variation, different imaging conditions, etc. In addition, these methods are very sensitive to how leaves are arranged (i.e. plant topology). Due to the intricacies of the learned models, such approaches cannot fully accommodate the variability of leaf appearance and arrangement not seen during training.

Therefore, it remains of interest to identify methods that can learn robust leaf counting predictors without the need for such sophisticated annotations. Giuffrida *et al*. (2015) and Pape and Klukas (2015b) made the observation that elementary cues in the image could relate to plant leaf count. A predictor can thus be built by first extracting the cues (features) from images and then relating them to the corresponding total leaf count. In particular, Pape and Klukas (2015b) used hand‐designed geometric features from the per plant segmentation mask to learn a relationship (a regression) between such features and leaf count. This approach required expert knowledge of the appropriate geometric features to use. On the contrary, the method of Giuffrida *et al*. (2015) uses *K*‐means (Coates *et al*., 2011), instead of hand‐designed features, to learn a visual dictionary from the data in a context‐adaptive fashion without expert knowledge.

Recently, deep neural networks have also been employed to address the leaf counting problem. These approaches essentially combine the task of finding suitable image features with the task of learning a good regression model relating the features to leaf count (Aich and Stavness, 2017; Dobrescu *et al*., 2017a; Ubbens and Stavness, 2017). These approaches show significant promise, but each of these is specialized: a new model and network for each plant species or cultivar, imaging condition, etc is required. In addition, all three approaches use only optical images, whereas different imaging sensors such as NIR or fluorescence (FMP) are now also commonly employed in plant phenotyping (Fiorani and Schurr, 2013; Klukas *et al*., 2014; Apelt *et al*., 2015; Gehan *et al*., 2017) .

In this paper, we introduce the Pheno‐Deep Counter (briefly PhenoDC), a multi‐input deep network that combines information coming from different imaging sources (termed *modalities* hereafter) to count the number of leaves of rosette‐shaped plants. In contrast to other approaches, we aim to build a single unified model that can be used for a variety of plants and imaging scenarios where plants are seen from the top in a laboratory setting. Critically, we demonstrate that by agglomerating data from a variety of sources the model learns better (deep learning algorithms require large numbers of data; Sun *et al*., 2017). Our approach also significantly enhances utility, as the same model can be used in a variety of scenarios and can be easily adapted for this purpose.

The main contributions of this work are:

- Multi‐modal model: an architecture that benefits from, and can use, multiple imaging modalities, for example classical color (RGB) and NIR images. We show that by combining information coming from multiple modalities, PhenoDC improves leaf count prediction. As an example, training our network with RGB images alone, PhenoDC predicts the correct leaf count in 55% of cases. Adding other modalities (e.g. NIR and FMP), the prediction accuracy increases to 88%.
- Ease of adaptation to new settings: our model can easily be adapted to work with another imaging setup (still assuming a top view), either by simply specializing the network for the new task or performing data agglomeration. We show that with a of handful plant images (regardless of the species tested) our network can be trained to count leaves for the new scenario. We showcase several experiments using images of *Arabidopsis thaliana* plants, as well as other plant species such as tobacco and komatsuna (a Japanese vegetable).
- State‐of‐the‐art performance: our approach can predict the number of leaves in unseen images with an error of ±1 leaf in about 80% of cases as compared with 57% in Giuffrida *et al*. (2015), further closing the gap to achieving human‐level performance (Giuffrida *et al*., 2018). This improves further when multi‐modal learning is used.
- Nocturnal leaf counting: we show that our network is also capable of counting leaves during the night by using NIR images, extending the applicability throughout the diel cycle, a feature not yet addressed by any other methods.

We perform a comprehensive analysis and comparison with other methods using a variety of data sources (both in‐house and publicly available). To aid adoption of our approach, we release code and trained models to allow plant scientists to utilize them in their experiments. This work also includes several experiments and discussion points to help elucidate how one can adopt such an approach (e.g. how many annotated samples are required and how to collect annotations) and how to interpret findings.

## Results

To showcase the performance of our approach, we employed four different datasets:

- A special collection from the PRL dataset (Minervini *et al*., 2016) and Aberystwyth dataset (Bell and Dee, 2016) that was used in the latest CVPPP 2017 Leaf Counting Challenge (LCC)^1^; it contains five different sub‐datasets (cf. Table S1 in the online Supporting Information). These datasets contain RGB color images of four different plant experiments, using different plants (and different cultivars), growth conditions and camera settings.
- The multi‐modality imagery database for plant phenotyping (Cruz *et al*., 2016), containing images of *A. thaliana* Col‐0 acquired in three different modalities (RGB, NIR, FMP);
- The RGB images in the komatsuna dataset (Uchiyama *et al*., 2017);
- Nocturnal Arabidopsis plant images acquired using a NIR camera (Dobrescu *et al*., 2017b).

Visual samples of these datasets are shown in Figure S1, whereas technical details are reported in Table S1.

**Figure 1:**
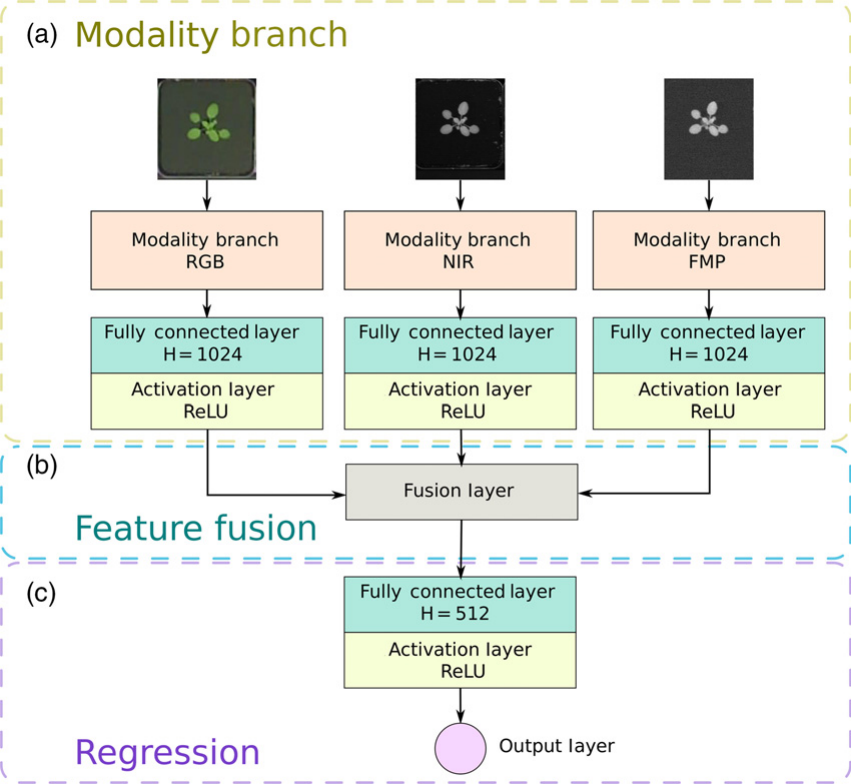
Schematic of the proposed deep architecture. (a) A modality branch, consisting of ResNet50 (He *et al*., 2016), extracts modality‐dependent plant features as a feature vector of 1024 neurons (RGB, visible light; NIR, near infrared; FMP, fluorescence). (b) The fusion part combines those features to retain the most useful information from each modality. (c) The regression part relates fused information with leaf count as a non‐linear regression. (This figure is best viewed in color online.) [Colour figure can be viewed at http://wileyonlinelibrary.com].

Our deep neural network, shown in Figure 1 and detailed in the Experimental Procedures, has been designed with the aim of accommodating inputs of variable size. To achieve this, our architecture breaks down the task of counting into several sub‐tasks. First, each image goes through a network that aims to find a fixed length vector representation to better describe a plant image. This is achieved by a sub‐network (*modality branch*), where each input source is processed independently. However, during training the network learns what can be usefully retained from each modality, which results in an image descriptor (a vector per image) that jointly represents all the useful information. Multi‐modal plant representation is accomplished by the *feature fusion* part of the architecture (details in Experimental Procedures). Finally, the fused image descriptor is related to leaf count by learning the parameters of a non‐linear regression model between the descriptor and leaf count. After the network has been trained, evaluation of a plant's image(s) (the plural is used to denote the presence of different modalities) provides an estimate of the leaf count.

To quantitatively assess the performance of our approach, we adopt the same evaluation metrics as in Giuffrida *et al*. (2015) (now a consensus in the broad community):

- Difference in count (DiC): mean and standard deviation of the differences between predicted leaf counts and ground truth (best value when mean and standard deviation are close to 0);
- Absolute difference in count (|DiC|): similar to before, but the differences between prediction and ground truth are absolute values (best value when mean and standard deviation are close to 0);
- Mean squared error (MSE): mean of the squared differences between prediction and ground truth (best value near to 0);
- Percentage agreement (%): number of times (as a percentage) that the predicted leaf count is exactly correct (best value at 100%).

Technical details about our deep architecture are provided in the Experimental Procedures, whereas evaluation metrics are detailed in Methods S1.

We present a comprehensive set of experiments that demonstrate the reliability of PhenoDC for leaf counting. To train our model, data are split into (at least) two datasets, namely a training and a testing set. The training set is needed to optimize the set of parameters specifying our model (see Experimental Procedures for further details). The testing set is required to evaluate the performance of the algorithm, using *unseen* data.

In the following, in a series of experiments we show:

- the benefit of data agglomeration across different sources;
- the superior prediction performance in the recent benchmark CVPPP 2017 dataset;
- that prediction error reduces when using multimodal sources with the dataset of Cruz *et al*. (2016); and lastly
- a set of experiments that demonstrate the flexibility of our network to adapt to other contexts, such as different plant species.

### Proof of concept: data agglomeration helps

Herein we aim to show that increasing data diversity in fact improves accuracy.

We isolated the A1 set of images in the CVPPP 2017 dataset (Minervini *et al*., 2016), which includes 128 images of *A. thaliana* Col‐0 for training. We followed the training procedure of Dobrescu *et al*. (2017a), assessing the performance of our network using a fourfold cross‐validation, randomly splitting the training set by the following proportions: (i) 64 images for learning; (ii) 32 images for validation; and (iii) 32 images for testing. The validation set allows us to monitor model performance during training and prevents overfitting (the case where the model has essentially memorized the training set and therefore cannot adapt to new data).

Using this learning protocol, the fourfold cross‐validation results are as follows:

- DiC −0.81 (0.85);
- *|*DiC*|* 0.94 (0.70);
- MSE 1.38;
- percentage agreement 25%.

We proceeded to add more data drawn from the CVPPP 2017 dataset, namely the A2 (*A. thaliana* of five genotypes), A3 (tobacco) and A4 (*A. thaliana* Col‐0) sets of images. As we continued to add data, we observed that the MSE reduced by about 50% (MSE 0.72). A similar improvement was seen in the percentage agreement, which increased to 56%. Finally, we wanted to evaluate which areas of an image contribute to the count. Ideally, the count produced by the network should only be influenced by regions of the image that contain plant. This analysis was performed using the method in Dobrescu *et al*. (2017a). We describe this analysis in Methods S2 and show the evaluation in Figure S2 on sample images taken from the CVPPP 2017 dataset.

This experiment highlights the benefit of data agglomeration, even when the sources are diverse. Since deep networks can form very complex functions (between input and output) the more data the better, and being ‘universal’ is better than being specialized (e.g. one model per plant species) as it reduces the chance of memorization.

### Evaluation and comparison with the state of the art on the CVPPP 2017 benchmark dataset

In this experiment we assess the performance of our network when trained on the heterogeneous CVPPP 2017 plant dataset and how it compares with state‐of‐the‐art methods in the literature.

**Figure 2:**
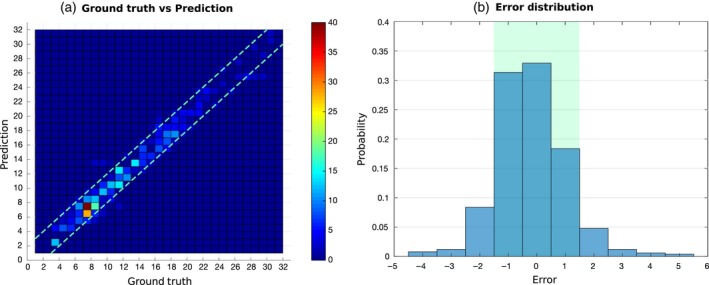
Leaf count prediction in the CVPPP dataset (all images together). (a) Ground truth versus prediction, shown as a scatter plot. Due to integer values the colors show how many points are overlapping. Dashed parallel lines show the ±1 leaf error range. Note that our approach has high agreement with the real leaf count. (b) Error distribution. Observe that there is 83% chance that the error will be ±1 within 0 (highlighted area), a number close to the agreement among human observers (about 90%; Giuffrida *et al*., 2018). (This figure is best viewed in color online.) [Colour figure can be viewed at http://wileyonlinelibrary.com].

We report quantitative results in Table 1, comparing our performance with other deep learning methods for leaf counting (Aich and Stavness, 2017) and leaf counting via segmentation (Romera‐Paredes and Torr, 2016; Ren and Zemel, 2017), as well as with the machine‐learning algorithm that won CVPPP LCC 2015 (Giuffrida *et al*., 2015). The CVPPP 2017 dataset contains as a subset data from previous competitions, allowing comparisons across the years and methods (but not on all data). Overall, PhenoDC outperforms all other methods, scoring the lowest MSE error in all datasets (1.56). Note that the single input model of our deep architecture achieved the best results on the CVPPP 2017 dataset in the LCC. A paired *t*‐test shows statistically significant gains when compared with Aich and Stavness (2017) (*P*‐value <0.0001; last column of Table 1). Figure 2 collates results across all images as: (i) the correlation between ground truth and prediction, showing the high agreement of our method (*R* ^2^ = 0.96); (ii) the distribution of error in leaf count, where it can be seen that in about 80% of cases the error is confined within the ±1 leaf range (for comparison Giuffrida *et al*. (2015) report 57% agreement for the same range).^2^ On some occasions PhenoDC might predict leaf counts incorrectly. Figure S3 shows some examples of such cases taken from the training set (Figure S3a, ground truth 20, predicted 17; Figure S3b, ground truth 18, predicted: 15; Figure S3c, ground truth: 13, predicted: 7). Overall, these images show several challenges to the network, including significant overlap and concentrated small leaves in the central part of the plant.

**Table 1:** Testing set results for PhenoDC trained on visible light (RGB) images from the CVPPP 2017 dataset (Scharr et al., 2014; Bell and Dee, 2016; Minervini et al., 2016). Difference in count (DiC) and absolute DiC (|DiC|) are given as mean and standard deviation (in parenthesis), with lower values being better. For the mean squared error (MSE) a lower value is better, while for percentage agreement (%) a higher value is better

		A1	A2	A3	A4	A5	All^a^
DiC^d^							
	**PhenoDC (this paper)**	**−0.39 (1.17)**	**−0.78 (1.64)**	**0.13 (1.55)**	**0.29 (1.10)**	**0.25 (1.21)**	**0.19 (1.24)**
	Giuffrida *et al*. (2015)	−0.79 (1.54)	−2.44 (2.88)	−0.04 (1.93)	–	–	–
	Romera‐Paredes and Torr (2016)	0.20 (1.40)	–	–	–	–	–
	Aich and Stavness (2017)	−0.33 (1.38)	−0.22 (1.86)	2.71 (4.58)	0.23 (1.44)	0.80 (2.77)	0.73 (2.72)
|DiC|^d^							
	**PhenoDC (this paper)**	**0.88 (0.86)**	1.44 (1.01)	**1.09 (1.10)**	**0.84 (0.76)**	**0.90 (0.85)**	**0.91 (0.86)**
	Giuffrida *et al*. (2015)	1.27 (1.15)	2.44 (2.88)	1.36 (1.37)	–	–	–
	Romera‐Paredes and Torr (2016)^b^^,^ c	1.10 (0.90)	–	–	–	–	–
	Ren and Zemel (2017)^b^^,^ c	0.80 (1.10)	–	–	–	–	–
	Aich and Stavness (2017)	1.00 (1.00)	**1.56 (0.88)**	3.46 (4.04)	1.08 (0.97)	1.66 (2.36)	1.62 (2.30)
MSE^d^							
	**PhenoDC (this paper)**	**1.48**	**3.00**	**2.38**	**1.28**	**1.53**	**1.56**
	Giuffrida *et al*. (2015)	2.91	13.33	3.68	–	–	–
	Aich and Stavness (2017)	1.97	3.11	28.00	2.11	8.28	7.90
%^e^							
	**PhenoDC (this paper)**	**33.3**	11.1	**30.4**	**34.5**	**33.2**	**32.9**
	Giuffrida *et al*. (2015)	27.3	**44.4**	19.6	–	–	–
	Aich and Stavness (2017)	30.3	11.1	7.1	29.2	23.8	24.0

a A paired *t*‐test between our method and Aich and Stavness 2017 (the only two approaches from the CVPPP Workshop 2017) shows statistically significant differences (*p* value <0.0001).

b Trained on A1 only.

c Training and inference are performed using per‐leaf segmentations and not total leaf count as with the other methods.

d Best values are those closer to 0.

e Best values are those closer to 1 (i.e. 100% in the case of Percentage Agreement).

Entries in bold represent the best performance.

In conclusion, PhenoDC is more reliable in terms of leaf counting than the current state‐of‐the‐art approaches.

### Multiple modalities and leaf counting

In this section we assess whether our network benefits from multi‐modal learning, leading to improved leaf count predictions.

For this experiment we used the dataset of Cruz *et al*. (2016), which contains images of *A. thaliana* wild‐type Col‐0 acquired using multiple sensors. Cruz and collaborators used 16 plants for 9 days, acquiring top‐view images from 9 a.m. to 11 p.m. (15 frames a day). This setup produced a dataset containing 2160 individual images altogether, albeit only 576 of them are annotated (images taken at 9 a.m., 12 p.m., 4 p.m. and 8 p.m.). Images were taken simultaneously in the following modalities: RGB, FMP, NIR and depth. The multiple sensors acquired the same plants simultaneously. Due to the heterogeneity of such sensors and their placement, image resolution (and effective image size) and alignment vary. We excluded depth images due to their extremely low resolution (about 30 × 30 pixels) compared with the others (see Table S1). Image samples are shown in Figure S1 with dataset details reported in Table S1. We randomly split the labeled dataset into three parts (50% training, 25% validation, 25% testing) and trained our network using fourfold cross‐validation.

To establish a baseline for our multi‐modal results and to find the most useful single modality (for the counting task), we first trained our network using only one of the available modalities as input at a time prior to using all modalities. As reported in Table 2, we obtained the best single‐input result using the NIR images (MSE = 0.39). This is due to the fact that NIR images in this dataset are sharper and more detailed. To demonstrate this, we visualize the activations produced by our network for each of the modality branches. Figure S4 shows the output of the first residual block (He *et al*., 2016) for three sample plants of the dataset (mean activation across the feature maps). Overall, most of the activations are focused on the region where the plant is located. Note that while some pixels are active on the background on RGB or FMP, the IR activations are mostly dominant on the plant, which demonstrates the benefit of using multi‐modal information. We obtained the best performance when all three inputs were used simultaneously: MSE was reduced by more than 50% and percentage agreement increased by about 19%.

**Table 2:** Testing the performance of PhenoDC on the multi‐modal dataset (Cruz *et al*., 2016). We report results when the network is trained using only a single modality and when also using all the three modalities

Training on	DiC^a^	|DiC|^a^	MSE^a^	%^b^
RGB only	0.02 (0.75)	0.48 (0.57)	0.56	55.7
FMP only	−0.06 (0.72)	0.45 (0.56)	0.52	58.7
NIR only	0.13 (0.61)	0.33 (0.53)	0.39	69.6
All (RGB, FMP, NIR)	0.11 (0.40)	0.13 (0.39)	0.17	88.5

DiC, difference in count; |DiC|, absolute DiC; MSE, mean squared error; %. percentage difference; RGB, visible light; FMP, fluorescence; NIR, near infrared.

a Best values are those closer to 0.

b Best values are those closer to 1 (i.e. 100%).

We conclude that combining information coming from multiple modalities improves counting accuracy. The fusion layer learns (cf. Figure 1b) to retain the most useful image features coming from any of the modality branches (cf. Figure 1c). These experiments highlight that multi‐modal learning can be useful for plant phenotyping purposes, and that our architecture can handle any number of inputs.

### Evaluation of network adaptivity capabilities

In this section we address the problem of how one can use PhenoDC by adapting to other experimental setups different from the one used during training.

We rely on the principle of fine‐tuning a *pre‐trained* network to significantly reduce the number of new training examples required to adapt the network (Bengio, 2012) and increase performance (Sun *et al*., 2017). Fine‐tuning entails the labeling of just a few images and using them to update the parameters of a network that has been pre‐trained to solve the same task but in a different context (e.g. for different plant species).

We demonstrate this capability in three different cases using the following datasets: tobacco plants (A3) from *Minervini et al*. (2016), komatsuna plants from *Uchiyama et al*. (2017) and other Arabidopsis cultivars using night‐time images *Dobrescu et al*. (2017b). (Further details of all these image datasets are given in Table S1 and Figure S1.) For these experiments, we first pre‐trained our neural network using only the Arabidopsis plant images A1, A2 and A4 in the CVPPP 2017 dataset (Bell and Dee, 2016; Minervini *et al*., 2016). This training dataset containing Arabidopsis plants, as reported in Table S1, does not include a large number of images, making the learning process challenging. The following experiments were also aimed at assessing the number of training images required to adapt the network to another scenario.

#### Tobacco plants (different species, imaging camera and settings)

**Figure 3:**
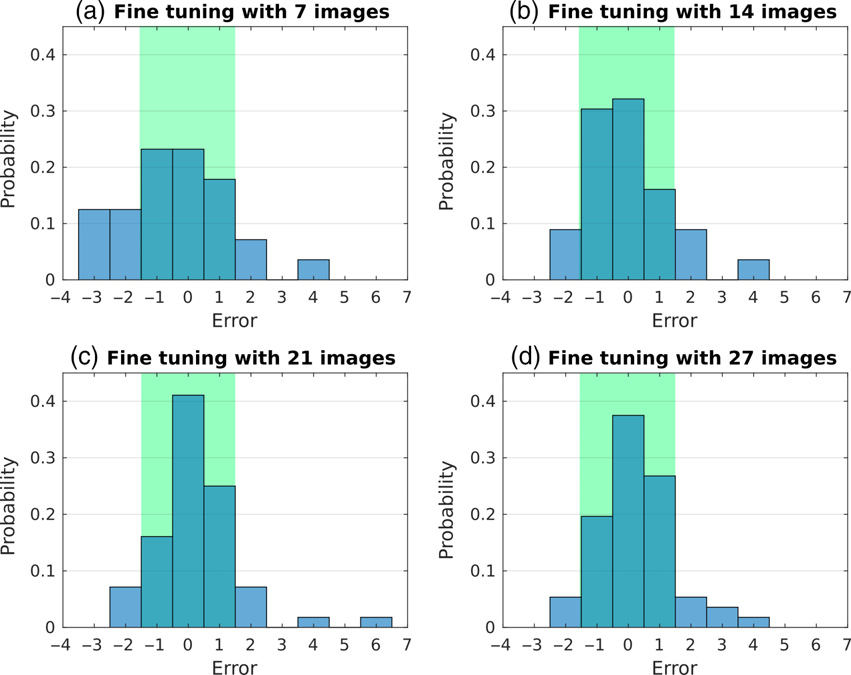
Error distribution of our network fine‐tuned using tobacco plants in the A3 dataset (Minervini *et al*., 2016). We reported the distribution of the error committed in the testing set, after refining the network parameters with 7 (a), 14 (b), 21 (c) and 27 (d) tobacco plants. When we train with more images (≥21), the highlighted area (error up to ±1 leaf, cf. Figure 2) contains more than 80% of the cases. (This figure is best viewed in color online.) [Colour figure can be viewed at http://wileyonlinelibrary.com].

We fine‐tuned the pre‐trained network using a variable number of tobacco training images. Specifically, we selected 7, 14, 21 and then 27 images to fine‐tune the pre‐trained network. The results of these experiments are reported in Table 3. Overall, we observed that more training data leads to better predictions in the testing set. As expected, the lowest error is obtained when we use all 27 images for training (MSE = 1.50). Figure 3 shows the distribution of the error that we registered during progressive learning. As more images are used, the error distribution narrows around 0. In fact, in about 80% of the data in the testing set our method is within 1 leaf error from the ground truth (highlighted areas in Figure 3), thus achieving more accurate predictions. Hence, we can conclude that after fine‐tuning with a handful of images (≥21 in this setup), PhenoDC can produce a reliable leaf count.

**Table 3:** Adapting (fine‐tuning) the parameters of the proposed architecture to work on tobacco images [A3 dataset (Minervini *et al*., 2016)] previously pre‐trained with Arabidopsis plants [A1, A2, and A4 (Bell and Dee, 2016; Minervini *et al*., 2016)]. We progressively increase the number of training images to find a suitable number of images required to create a meaningful model that can count tobacco leaves. The table reports the results on the held‐out testing set

No. of training images	DiC^a^	|DiC|^a^	MSE^a^	%^b^
7	−0.39 (1.65)	1.32 (1.07)	2.83	23.2
14	0.00 (1.32)	0.96 (0.90)	1.75	32.1
21	0.27 (1.36)	0.87 (1.07)	1.91	41.1
27	0.25 (1.20)	0.86 (0.87)	1.50	37.5

DiC, difference in count; |DiC|, absolute DiC; MSE, mean squared error; %. percentage difference.

a Best values are those closer to 0.

b Best values are those closer to 1 (i.e. 100%).

#### The komatsuna case (different species, imaging camera and settings)

This dataset contains 300 RGB images of five different komatsuna plants, six images/day for 10 days (images were taken every 4 h from 3 p.m. until 3 p.m. the following day). We split the dataset as follows (see Table S1):

- training set: two plants (IDs 00 and 01), entire timeline (120 images);
- validation set: one plant (ID 04), entire timeline (60 images);
- testing set: two plants (IDs 02 and 03), entire timeline (120 images).

We fine‐tuned our pre‐trained network by progressively increasing the size of the training set to 10, 20, 30 and then 40 images per plant, choosing time frames that followed the evolution of plant growth. Overall, the results in Table 4 show that more data contribute to more accurate results. Predictions become very accurate when 40 images per plant are used during training, showing a reduction of the MSE by 50%, compared with training using 10 images per plant.

**Table 4:** A similar process to that described in Table 3 but repeated for komatsuna plant leaf counting based on data available in Uchiyama *et al*. (2017). The model has been trained on Arabidopsis as described in Table 3. Results shown refer to the testing set

No. of training images per plant	Hours of the day	DiC^a^	|DiC|^a^	MSE^a^	%^b^
10	3 p.m.	−0.74 (1.08)	0.96 (0.89)	1.71	35.0%
20	3 p.m., 11 a.m.^c^	−0.54 (0.95)	0.86 (0.65)	1.19	25.0%
30	3 p.m., 3 a.m.^c^, 11 a.m.^c^	0.18 (0.92)	0.67 (0.66)	0.88	44.2%
40	3 p.m., 3 a.m.^c^, 7 a.m.^c^, 11 a.m.^c^	0.24 (0.84)	0.59 (0.64)	0.76	49.1%

DiC, difference in count; |DiC|, absolute DiC; MSE, mean squared error; %, percentage difference.

a Best values are those closer to 0.

b Best values are those closer to 1 (i.e. 100%).

c Images taken on the following day.

#### Nocturnal images of Arabidopsis plants (different cultivars, settings and modality)

Night images are usually acquired using infrared cameras and specific LED lights that illuminate the scene with NIR (a wavelength of 940 nm which does not alter natural plant development; Cruz *et al*., 2016; Dobrescu *et al*., 2017b). We selected and annotated a subset of night images (from Dobrescu *et al.,* 2017b). Specifically, we selected 18 plants and sampled one image per night every other day for 8 days (a total of 72 images). Examples of nocturnal images are shown in Figure S1. We pre‐trained the network using the NIR images from Cruz *et al*. (2016) and fine‐tuned it using 10 plants for training (40 images in total), 4 plants for validation (16 images) and the last 4 for testing (16 images). Since these images come from different ascensions of *A. thaliana*, we randomly changed the training/validation/testing set four times. Quantitative results on the testing error are: DiC, −0.14 (0.77); *|*DiC*|*, 0.52 (0.59); MSE, 0.61; percentage agreement, 53.1%. Overall, the error is very low (MSE < 1), demonstrating the utility of our machine‐learning approach to leaf counting during the night.

To summarize, these experiments demonstrated that PhenoDC can adapt to different scenarios of considerable complexity. Acceptable performance can be attained using just a few images (e.g. 14 in the case of tobacco). In addition, by fine‐tuning our network with Arabidopsis images acquired during the night, plant growth analysis during the entire circadian cycle is allowed (Apelt *et al*., 2015).

## Discussion

In this paper we report on PhenoDC, a deep artificial neural network that can predict the total number of leaves from top‐view plant images. We have shown the effectiveness and reliability of our network architecture using several plant datasets. Specifically, we show that data agglomeration helps to improve accuracy: as more datasets were added the MSE fell by 50%. A similar error reduction was  also observed when the network was trained with multi‐modal data, showing that combining information from multiple imaging sources helps to train a better regression model and allows learning of better features. We showed that our method can adapt to new settings and demonstrated that a refinement step, fine‐tuning, can be used to achieve excellent performance even with only a few images for training. We also demonstrate that NIR modalities can be used to count leaves during darkness, permitting leaf counts for detailed plant growth analysis throughout the circadian cycle.

Our approach to leaf counting involves learning of meaningful image features across all modalities and then relates features to leaf count via non‐linear regression. We train both aspects together, thus adapting image features while learning the regressor. This has been central to the success of deep learning in a variety of problems, from image recognition to self‐driving cars (LeCun *et al*., 2015). Furthermore, our approach offers a single model to solve the same task for any input. Our robust and accurate neural network can be extended for new input/modalities without changing the overall architecture. This simplifies adoption and permits the sharing of model updates when new experiments have been made available on the basis of our architecture. Therefore, by placing our pre‐trained PhenoDC and source code (and instructions) into the publicly available repository at https://bitbucket.org/tuttoweb/pheno-deep-counter, we hope to accelerate the adoption of such methods in plant phenotyping analysis.

Our network was evaluated on top‐down views of dicot rosette‐shaped plants. Clearly, this is one setup among many others. It is possible, though, that an *ideal* leaf counting algorithm would also be able to work on monocots, and even tree canopies with thousands of leaves, given enough training data. Unfortunately, we currently lack such curated datasets with these scenarios and are unable to experimentally assess how PhenoDC would perform, although it still brings us a step closer towards generalization.

In this work we focused on ‘*how many*’ rather than ‘*which*’ annotated images are needed to train a good regression model. It goes without saying that adequate image resolution and quality are necessary. Generally, images that show appearance diversity are good images to annotate. In a time‐lapse setting, images spanning a set interval of the time series would be a good start. However, better approaches exist to find the best set of images to jointly inform the model, known as active learning. Active learning with neural networks is an ongoing research problem in machine learning. We have previously shown, using plant descriptors and data mining (He, 2016), promising potential in identifying images for annotation.

Furthermore, this work assumed that ground‐truth annotations (provided by expert observers) can be considered as gold standard and error‐free. However, it is widely known (e.g. in applications in medical image analysis) that even expert observers show variation. Recently, several related works have shown that variations exist among annotators in labeling plant images (Giuffrida *et al*., 2018) or in assigning specific (a)biotic plant stress via visual inspection of leaf blades (Ghosal *et al*., 2018). Interestingly, intra‐ and inter‐observer variation can also be used to assess algorithm performance. Based on the findings of Giuffrida *et al*. (2018), inter‐observer variation has a MSE of 0.81 [inexperienced annotators on a subset of Arabidopsis images used in Minervini *et al*. (2017)]. Experienced and inexperienced annotators are within the ±1 leaf error range in about 90% of cases, whereas PhenoDC is within ±1 leaf error in about 80% of cases, thus bringing us closer to human‐level performance.

Evidently, ‘*true*’ ground‐truth data can only be attained by aggregating observations from many annotators to reach a consensus. Since doing this with experts is time‐consuming, recent studies using dedicated online platforms, such as Zooniverse, can alleviate this problem by tapping into the power of citizen scientists. An alternative is to use simulated or synthetic data, where ground truth is absolute by design. Simulated data have recently been used in the plant community to count the number of fruits (Rahnemoonfar and Sheppard, 2017) and the number of leaves in Arabidopsis plants (Mündermann *et al*., 2005; Ubbens *et al*., 2018). Simulated images are provided by software that takes object parameters as input (e.g. plant age, number of leaves). Although images may lack visual realism, recent innovations in image synthesis (Giuffrida *et al*., 2017) point to the potential of creating synthetic images of realistic appearance.

In conclusion, we present a deep learning approach to leaf counting with a neural network. Trained with examples of images and corresponding plant leaf counts, our approach can achieve outstanding results in a variety of settings. Our model handles many input modalities and has been tested with images of different species, cultivars and also with images at night. By making it openly available to the community we hope that it will stimulate large‐scale analysis in plant phenotyping of a crucial plant trait – leaf count – and help relieve the analysis bottleneck (Minervini *et al*., 2015a).

## Experimental procedures

In this section we discuss technical details of the deep network architecture that characterizes our PhenoDC, shown in Figure 1. We optimize all computational blocks simultaneously to obtain a mapping between input images and leaf counts. For our purposes, we used up to three inputs: RGB, NIR and FMP.

### Modality branch

The modality branch is a sub‐network that processes each input (see Figure 1a). We used the ResNet50 architecture (He *et al*., 2016), as in Dobrescu *et al*. (2017a). Each input is processed independently of the others and generates a vector representation specific to its input, ensuring meaningful and discriminative features. Each branch ends with a fully connected layer of 1024 neurons using rectifier (ReLU) non‐linearity, which allows the suppression of negative values during the process of feature extraction. Each input results in an output vector of the same size independent of the input image size.

### Feature fusion

Feature fusion is the process that combines information coming from all modalities to retain the most meaningful features. Following the concept in Chartsias *et al*. (2018), we apply an element‐wise maximum fusion layer. We display this segment of the network in Figure 1(b).

### Regression

Regression is the process of relating fused information to leaf count (cf. Figure 1c). The output of the fusion layer is given to another fully connected layer of 512 neurons with ReLU activation function. At the end of the network, the output of the last layer is given to a single neuron that makes the actual prediction of the number of leaves. During training, we minimize the MSE between predicted leaf count and ground truth. The model predicts real numbers and we round the leaf count to the nearest integer only at the test time.

### Training strategies

We employ three common training strategies to improve network training and performance. First, we initialize our network with pre‐trained parameters (rather than random ones), computed previously based on an image recognition task (Russakovsky *et al*., 2015). Second, we use an *L*_2_ regularizer in the last fully connected layer before the output (i.e. the regression component). This technique prevents the network from learning large weights which may produce unstable results. For all experiments in this paper we set this regularization constant to λ *= *0.02. Finally, to artificially increase robustness to changes in view (rotation, translation and camera position), we perform *dataset augmentation* during training. Specifically, we apply random geometrical transformations to the training data (e.g. random rotations, zoom‐ins, shifts); this helps the network to learn from more data without having to collect more data. Our network was trained using a learning rate of η = 0.0001.

### Validation set

One of the problems arising in network training is when to stop training. The typical approach in machine learning is to also use a small set of labeled data, called the validation set (Theodoridis and Koutroumbas, 2008). We therefore used an early stop criterion to interrupt the learning procedure; terminating the training after 10 epochs we observed that the validation error had started to get worse.

### Image pre‐processing

While combining data across different sources (data agglomeration) has benefits, the images coming from different setups exhibit variations in intensity and size that need to be corrected. For instance, images in A1 (Minervini *et al*., 2016) and images in A4 (Bell and Dee, 2016) were acquired with different cameras and different illumination conditions, although they may show the same plant species (*A. thaliana* Col‐0). To ameliorate variations in illumination, we perform histogram normalization on all images and to standardize image size we resize all images of a modality to the same size of 320 × 320 pixels. For the multi‐modal images (Cruz *et al*., 2016), RGB images are too small to be provided to the RGB modality branch, as ResNet needs images of at least 200 × 200 pixels in size (cf. Table S1). In this case, we upsampled the images to 240 × 240 pixels whereas the images from the other modalities were left unchanged.

### Implementation details

We implemented our deep neural network using Keras (Chollet, 2015), an open‐source library for deep learning in Python, with a Tensorflow backend. We performed our training experiments in a machine with a TITAN X GPU. Note that such equipment is not necessary for fine‐tuning and adapting our network to new experimental data.

## Funding

This work was partially supported by BBSRC grant BB/P023487/1.

## Author contributions

MVG implemented the method and analyzed data; MVG and SAT planned the experiment; MVG, PD and SAT wrote the article; all authors read and approved the article.

## Conflict of interest

The authors declare no conflicts of interest.

## Supporting information


**Figure S1.** Sample images of the employed datasets.

**Figure S2.** Visualization of which part of an image contributes to the leaf counting.

**Figure S3.** Some examples of images taken from the CVPPP dataset where the leaf count prediction is inaccurate.

**Figure S4.** Visualization of the output of the first residual block for each of the modality branches.

**Table S1.** Details of the plant phenotyping datasets used in this paper.

**Methods S1**. Evaluation metrics.

**Methods S2.** Assessing what the network counts.

## Notes

1 More information at: https://www.plant-phenotyping.org/CVPPP2017-challenge.

2 It is relevant to point out that, unlike in our method, Giuffrida *et al*. (2015) used only the A1, A2 and A3 images. PhenoDC still has an accuracy of ±1 leaf range in about 80% of cases, when trained and tested on the same portion of the data to make fair comparisons.
