# Supplementary figures and images for: Pheno‐Deep Counter: a unified and versatile deep learning architecture for leaf counting
Source: Plant J. 2018 Sep 11;96(4):880–90. doi: 10.1111/tpj.14064 (PMC6282617; doi:10.1111/tpj.14064)

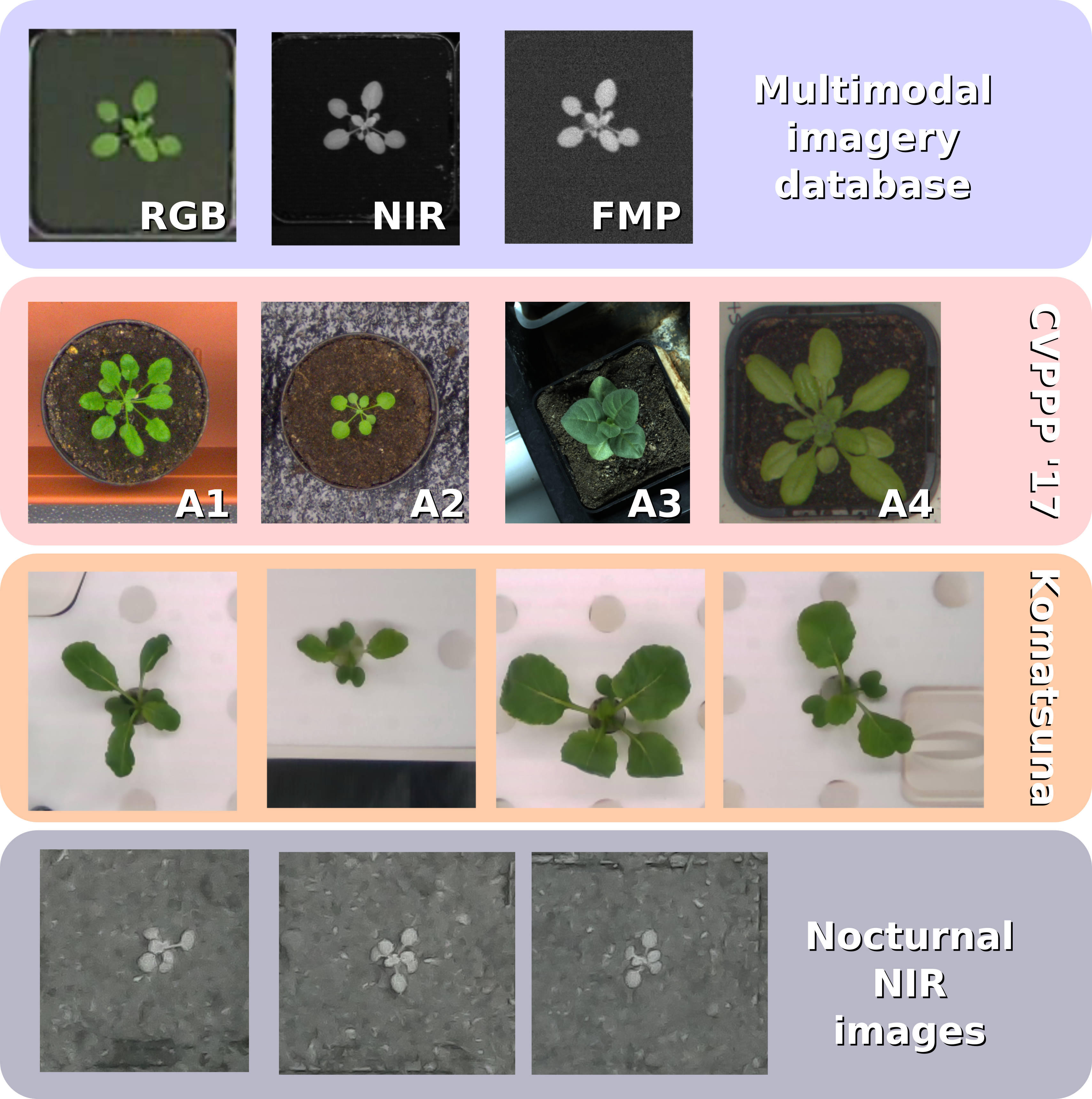

Supplement: Supplementary file 1 — Figure S1. Sample images of the employed datasets. [file TPJ-96-880-s001.png]

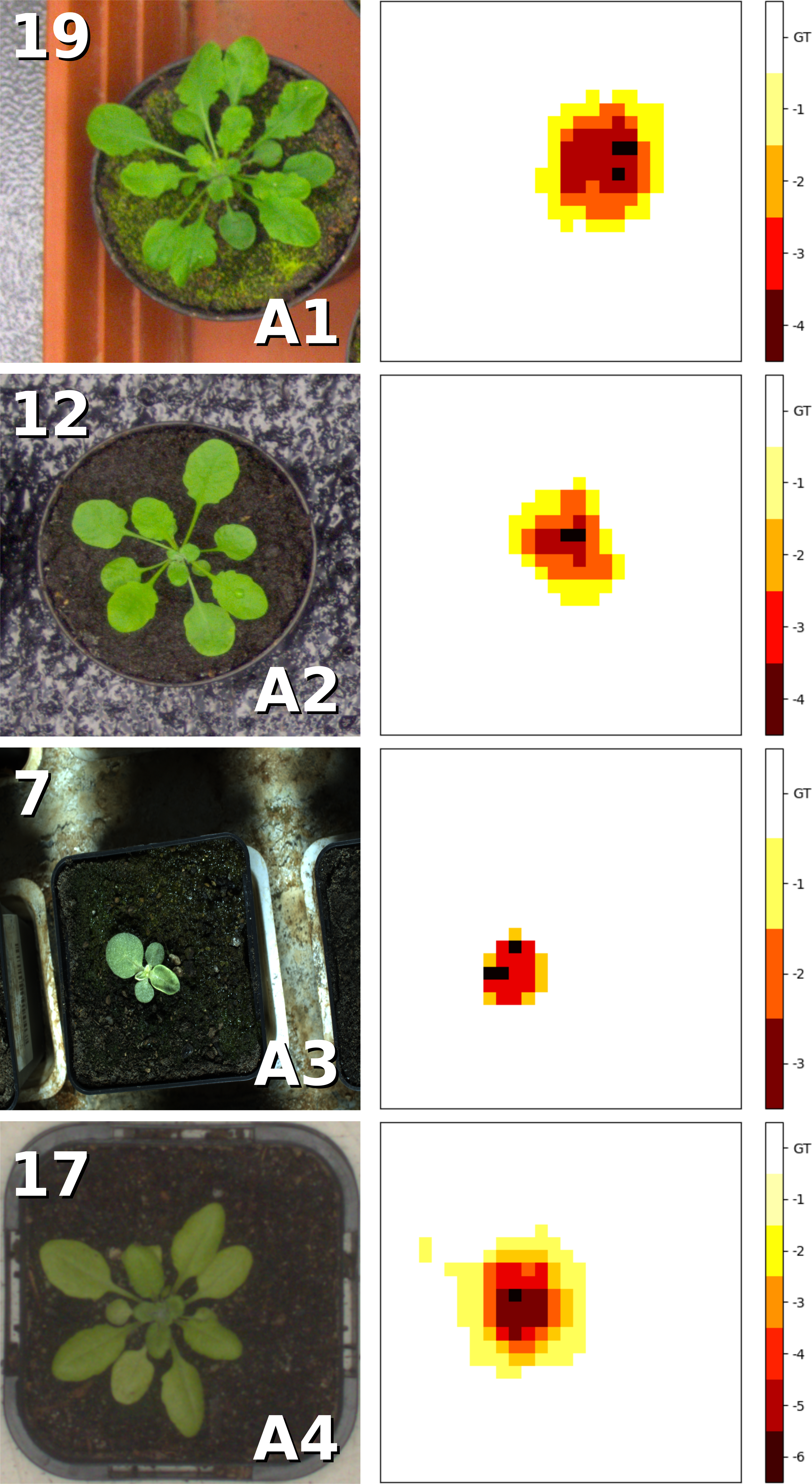

Supplement: Supplementary file 2 — Figure S2. Visualization of which part of an image contributes to the leaf counting. [file TPJ-96-880-s002.png]

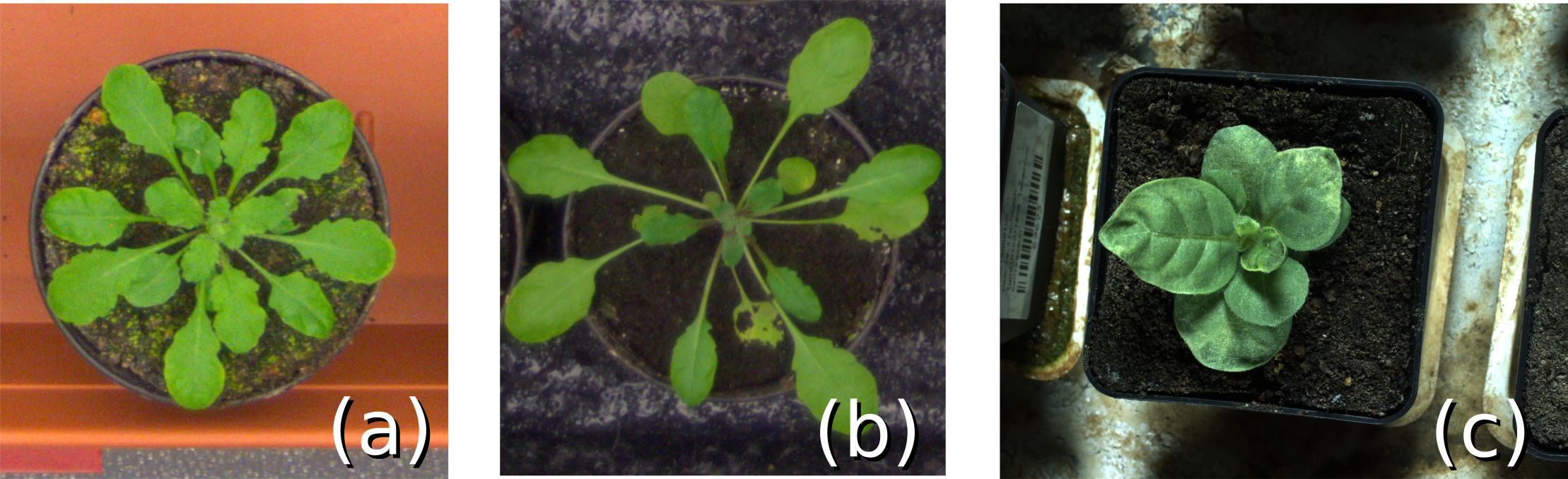

Supplement: Supplementary file 3 — Figure S3. Some examples of images taken from the CVPPP dataset where the leaf count prediction is inaccurate. [file TPJ-96-880-s003.png]

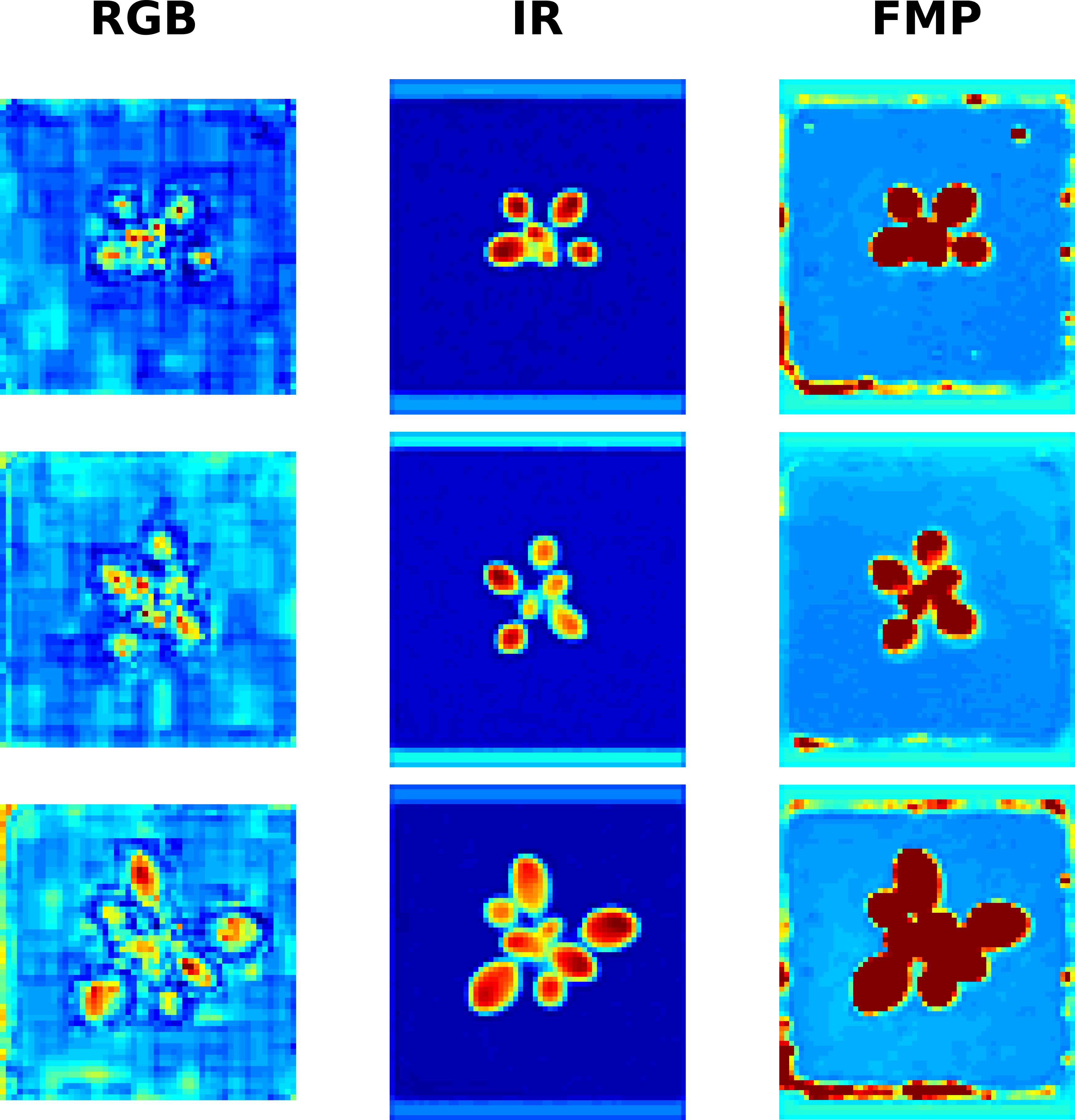

Supplement: Supplementary file 4 — Figure S4. Visualization of the output of the first residual block for each of the modality branches. [file TPJ-96-880-s004.png]
