# Supplementary material for: Pheno‐Deep Counter: a unified and versatile deep learning architecture for leaf counting
Source: Plant J. 2018 Sep 11;96(4):880–90. doi: 10.1111/tpj.14064 (PMC6282617; doi:10.1111/tpj.14064)
Supplement: Supplementary file 5 — Table S1. Details of the plant phenotyping datasets used in this paper. [file TPJ-96-880-s005.docx]

**Table S1.** Details of the plant phenotyping datasets used in this paper.

| *Dataset* | *Type of plants* | *Modalities* | *Training Images* | *Testing Images* | *Image Resolution* |
| --- | --- | --- | --- | --- | --- |
| CVPPP 2017 (Bell and Dee, 2016; Minervini et al., 2016; Scharr et al., 2014) | | | | |  |
| A1 | *Arabidopsis thaliana* Col-0 | RGB | 128 | 33 | $500\times530$ |
| A2 | *Arabidopsis thaliana* Col-0, ctr, pgm, ein2.1, adh1 | RGB | 31 | 9 | $530\times565$ |
| A3 | *Nicotiana tabacum* | RGB | 27 | 56 | $2448\times2048$ |
| A4 | *Arabidopsis thaliana* Col-0 | RGB | 624 | 168 | $441\times441$ |
| A5 | All of above | RGB | N/A | 235 | All of above |
|  | | *Total:* | *810* | *501* |  |
| Multi-modal imagery database for plant phenotyping (Cruz et al., 2015) | | | | |  |
|  | *Arabidopsis thaliana* Col-0 | RGB | 288 + 144 for validation | 144 | $120\times120$ |
|  |  | IR |  |  | $273\times273$ |
|  |  | FMP |  |  | $273\times273$ |
| Komatsuna (Uchiyama et al., 2017) | | | | |  |
|  | Komatsuna plants | RGB | 120 + 60 for validation | 120 | $480\times480$ |
| Arabidopsis thaliana nocturnal images from *Dobrescu et al., 2017b* | | | | |  |
|  | *Arabidopsis thaliana* Col-0, 1a2b, a13b | NIR | 40+16 for validation | 16 | $490\times460$ |
