# Supplementary material for: Pheno‐Deep Counter: a unified and versatile deep learning architecture for leaf counting
Source: Plant J. 2018 Sep 11;96(4):880–90. doi: 10.1111/tpj.14064 (PMC6282617; doi:10.1111/tpj.14064)
Supplement: Supplementary file 6 — Methods S1. Evaluation metrics. [file TPJ-96-880-s006.docx]

**Supplemental Methods S1 - Evaluation metrics**

To evaluate our deep neural network, we used the metrics in *Giuffrida et al., 2015*. Let $y_{i}$ be the ground-truth leaf count for the $i$-th image, and $\hat{y}_{i}$ be the output of a counting algorithm, we can define the difference in count as

$$\Delta_{i}=\hat{y}_{i}- y_{i}. (1)$$

From this definition, we can formalize the evaluation metrics as follows:
**Difference in count (*DiC*):** mean and standard deviation of Equation (1):

${DiC}_{\mu} =\frac{1}{N}\sum_{i=1}^{N} \Delta_{i}, (2a)$

$${DiC}_{\sigma}=\sqrt{\frac{\sum_{i=1}^{N} \left( \Delta_{i}-{DiC}_{\mu} \right)}{N-1}}, (2b)$$

where $N$ is the number of images in the dataset.

**Absolute Difference in count (*|DiC|*):** similar to (2a) and (2b), but the differences in (1) are taken in absolute value:

${|DiC|}_{\mu} =\frac{1}{N}\sum_{i=1}^{N} \left| \Delta_{i} \right|, (3a)$

$$|{DiC|}_{\sigma}=\sqrt{\frac{\sum_{i=1}^{N} \left( |\Delta_{i}|-{|DiC|}_{\mu} \right)}{N-1}.} (3b)$$

**Mean squared error (*MSE*):** mean of the squared differences in (1):

$MSE =\frac{1}{N}\sum_{i=1}^{N} \Delta_{i}^{2}. (4)$

**Percentage Agreement (*%*):** number of times (in percentage) that the ground-truth is equal to the prediction, namely the difference in (1) is 0:

$$\%=\frac{1}{N}\sum_{i=1}^{N} 1\left\{ \Delta_{i}=0 \right\}, (5)$$

where $1\{x\}$ is the *indicator function* that returns 1 if the predicate $x$ is true, 0 otherwise.

**Supplemental Methods S2 – Assessing what the network counts**

In this experiment, we evaluate if the network considers only the leaf area to perform counting. Differently from other state-of-the-art methods (e.g., Giuffrida et al., 2015; Aich and Stavness, 2017), we do not provide per-plant segmentation masks during learning and inference to our network. Therefore, one can question whether the network is actually counting leaves, or if the prediction is influenced by unrelated regions of the images, such as background.

To assess this, we employed the approach in *Dobrescu et al.* (2017a). Specifically, we mask part of the image with a 60×60 sliding window and see how this affects the leaf count. Ideally, when the window does not obscure parts of the plant (i.e. covers only the background), the leaf count should remain unchanged. In Figure S4, we display such an evaluation on a sample image of each of the four plant datasets in CVPPP 2017. We iteratively covered all possible locations of each image with this mask and observed that the largest contribution to the counting is very specific in the regions corresponding to plant leaves.
