# Supplementary material for: Pheno‐Deep Counter: a unified and versatile deep learning architecture for leaf counting
Source: Plant J. 2018 Sep 11;96(4):880–90. doi: 10.1111/tpj.14064 (PMC6282617; doi:10.1111/tpj.14064)
Supplement: Supplementary file 7 — Methods S2. Assessing what the network counts. [file TPJ-96-880-s007.docx]

**Figure S1.** Sample images of the employed datasets. *First row:* RGB, near-infrared, and fluorescence images of the same plant from the multi-modal imagery database for plant phenotyping (Cruz et al., 2015). *Second row:* images from the A1, A2, A3, and A4 datasets from CVPPP 2017 (Bell and Dee, 2016; Minervini et al., 2016; Scharr et al., 2014). *Third row:* samples of Komatsuna plants from *Uchiyama et al.* (2017). *Last row:* samples of nocturnal images of Arabidopsis plants in *Dobrescu et al.* (2017b). (Best viewed in color).

**Figure S2.** Visual diagram showing which part of a plant image contributes the most for the counting. We shift a 60×60 black patch entirely over a plant image and we show that areas corresponding to the plant gives the highest contribution to the count. In the top-left corner of each image we report the ground-truth (GT) leaf count of the plant.

**Figure S3.** Examples of plant images taken from the CVPPP dataset where our network predicts the inexact number of leaves. (a) GT: 20; predicted: 17. (b) GT: 18; predicted: 15. (c) GT: 13; predicted: 7.

**Figure S4.** Activations after the first residual block in the RGB, IR, and FMP modality branches. The output of this block layer consists of 256 feature maps. We display the mean for each pixel. (Best viewed in color).

**Table S1.** Details of the plant phenotyping datasets used in this paper.
